# VPS33B与GP1BA基因双重杂合变异致血浆VWF水平严重降低：1例报告及文献复习

**DOI:** 10.3760/cma.j.cn121090-20231216-00317

**Published:** 2024-06

**Authors:** 思倩 马, 霞 白, 丽娟 曹, 珍妮 马, 子轩 丁, 自强 余, 淼 江

**Affiliations:** 1 苏州大学附属独墅湖医院血液科，苏州 215028 Department of hematology, Dushu Lake Hospital Affiliated to Soochow University, Suzhou 215028, China; 2 国家血液系统疾病临床医学研究中心，苏州大学附属第一医院，江苏省血液研究所，苏州 215007 National Clinical Medical Research Center of Blood Diseases, The First Affiliated Hospital of Soochow University, Jiangsu Institute of Hematology, Suzhou 215007, China; 3 卫健委血栓与止血重点实验室，苏州 215006 Key Laboratory of Thrombosis & Hemostasis of National Health Commission of People's Republic of China, Suzhou 215006, China

## Abstract

一例28岁女性，孕期常规体检发现凝血因子Ⅷ活性（FⅧ∶C）<1％、血管性血友病因子抗原（VWF∶Ag）<1％。二代测序未发现其VWF基因外显子区域存在致病变异。由于该患者临床表现与Ⅲ型血管性血友病（VWD）临床特征不符，因此采用三代测序技术对该患者及其家系成员进行全基因组测序，发现该患者父系家族中有多位成员中携带VPS33B基因杂合变异c.869G>C，携带该变异的家系成员均有不同程度的VWF水平降低（39％～56％）。同时，先证者还检出GP1BA基因杂合变异c.1474dupA，ACMG及Clinvar数据库判断该变异与“血小板型假性VWD”相关。VPS33B基因杂合变异导致的VWF水平降低家系为国际首次报道，VPS33B基因与GP1BA基因双重杂合变异引起血浆VWF水平严重降低病例之前也未见报道。

## Abstract (en)

A 28-year-old woman was found to have coagulation factor Ⅷ activity（FⅧ∶C）<1％ and von Willebrand factor antigen（VWF∶Ag）<1％ during routine prenatal examinations. No pathogenic variation was found in the exon region of the VWF gene using next-generation sequencing. The clinical presentation of this patient does not match the clinical characteristics of type Ⅲ hemophilia［von Willebrand disease（VWD）］; therefore, third-generation sequencing technology was used to perform whole-genome sequencing on the patient and her family members. Multiple members of the patient's paternal family carried a heterozygous variant of VPS33B, c.869G>C. The family members carrying this variant all had varying degrees of reduced VWF levels（39％–56％）. Moreover, the proband was detected with the heterozygous variant c.1474dupA in GP1BA. The ACMG and Clinvar databases determined that this variation was associated with platelet-type pseudo VWD. The decrease in VWF levels caused by heterozygous variations in VPS33B in families is the first international report, and no previous studies have reported cases of severe decrease in plasma VWF levels caused by double heterozygous variations in VPS33B and GP1BA.

血管性血友病因子（von Willebrand factor, VWF）含量降低或者功能缺陷可导致不同程度的出血倾向，临床上称为血管性血友病（von Willebrand disease, VWD）[1]。VWF是一种存在血浆中的多聚糖蛋白，由血管内皮细胞与巨核细胞合成，其主要功能是介导血小板与血管损伤部位的黏附以及作为凝血因子Ⅷ（FⅧ）的载体以维持FⅧ在循环中不被过快降解。VWD作为一种最常见的遗传出血性疾病，其发病率在0.01％～1％[2]。根据目前文献，所有VWD均由VWF基因缺陷所导致，临床指南将因VWF基因缺陷引起的VWD分为3型[1]。此外，尚有一种VWF降低的情形并非由VWF基因缺陷导致，而是由于血小板膜糖蛋白Ⅰbα（GPⅠbα）变异，使VWF与GPⅠbα结合力过强，导致循环中VWF水平降低，该症被称为假性VWD，属于罕见的隐性遗传性血小板功能性疾病[3]。除此之外，在临床上未见到其他调控VWF基因表达或分泌的上下游基因变异引起VWF水平降低的报道。

VPS33B属于Sec1/Munc18蛋白（SM蛋白）家族，是转运蛋白家族成员之一[4]。VPS33B在血小板α-颗粒形成相关的囊泡形成与运输过程可能具有重要作用[5]，血小板α颗粒内包含VWF在内的多种黏附分子和凝血成分。本文报道一例罕见的由VPS33B联合GP1BA基因双重杂合变异引起血浆VWF水平严重降低的患者以及因VPS33B基因缺陷引起血浆VWF水平降低的家系。

## 病例资料

患者，女，28岁，孕25周。孕期常规体检发现凝血因子异常，查FⅧ活性（FⅧ∶C）<1％；VWF∶Ag<1％。自幼有鼻出血病史，每月1次，每次持续约10 min，按照国际血栓与止血学会出血评分工具（ISTH-BAT）评分为1分，儿童期较严重，成年后症状减轻；月经量增加，每次约5～7 d，对照月经失血图评分法为120分，有轻度缺铁性贫血，HGB 92 g/L，未进行补铁治疗；无其他出血表现。家族中无出血病史、无其他特殊病史。体格检查：骨骼、关节无畸形；未见皮肤瘀点瘀斑。实验室检查：VWF∶Ag<1％，VWF多聚物中超大分子量VWF多聚物较正常对照轻度减少（由于患者VWF含量极低，因此电泳样本采用与正常对照不同的稀释比例和上样量），大分子和中分子量VWF多聚物条带正常（图1A）；VWF抑制物阴性；PLT 125×10^9^/L，PFA-100 >300 s；ADP及胶原诱导血小板聚集功能正常；瑞斯托霉素诱导血小板聚集试验因患者VWF含量低未能测出，将患者血小板与正常人贫血小板血浆（PPP）混合后，以0.25 µg/ml浓度瑞斯托霉素诱导血小板聚集，实验结果显示聚集速率及2 min最大聚集率高于正常对照，但5 min最大聚集率与正常对照无差异（图1B）。肝肾功能正常，血型为B型。全外显子基因检测（WES）发现先证者VPS33B基因c.869G>C，p.Arg290Pro杂合变异。通过对其家族成员的基因测序和血液学分析，发现该患者父系家族中有多位成员中携带VPS33B基因杂合变异c.869G>C，并且携带该变异的家系成员均有不同程度的VWF水平降低（39％～56％）（表1）。该变异在各数据库中未见报道，其致病性未明，经变异预测软件PolyPhen-2、SIFT、Provean等分析预测该变异具有致病性；蛋白结构预测（http://toolkit.tuebingen.mpg.de）分析显示，VPS33B蛋白中氨基酸位点R290与E248、F286形成氢键，并且R290与D149之间可能存在非共价相互作用（图1C），R290P变异使该位点与周边氨基酸之间的结合被破坏，导致VPS33B与其配体结合的能力受到影响。同时，三代测序还检出先证者携带1处GP1BA杂合变异c.1474dupA，ClinVar数据库中收录有该变异与“血小板型假性VWD“相关性的报道（https://www.ncbi.nlm.nih.gov/clinvar/variation/2440587/），美国遗传学会（ACMG）将该变异导致“血小板型假性VWD“的致病可能性定义为5级中的第2级（Likely Pathogenic）。先证者的上述基因变异经Sanger测序得以验证（图1D）。根据先证者表型、基因突变-蛋白结构功能比对分析及家系遗传差异分析，考虑其为VPS33B基因联合GP1BA基因双重杂合变异引起假性重度VWD病例。家系图见图2。

**图1:**
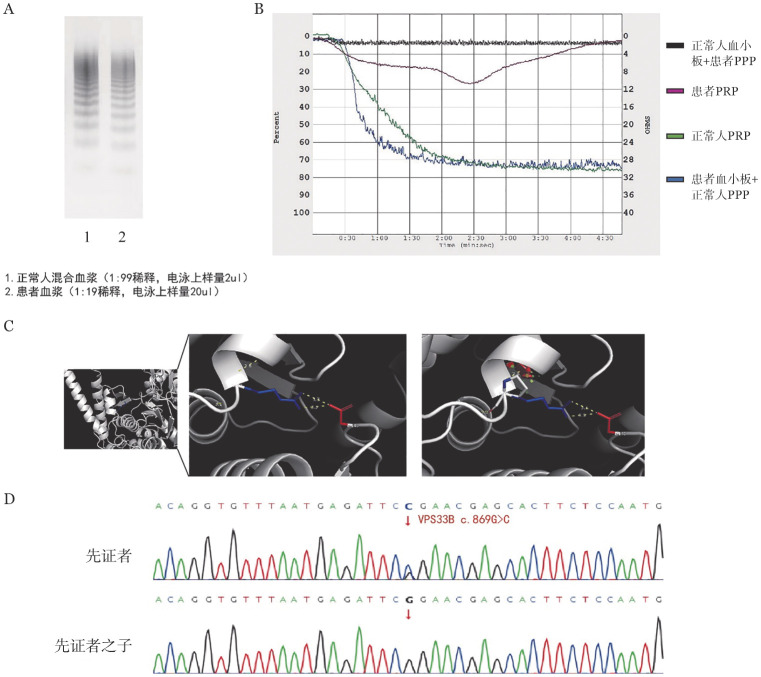
VPS33B基因变异家系实验室检测 **A** 血浆VWF多聚物电泳检测结果；**B** 血浆-血小板交叉检测瑞斯托霉素诱导血小板聚集；**C** 蛋白结构预测R290与VPS33B中其他残基的相互作用，R290P变异使该位点与周边氨基酸之间的结合被破坏；**D** Sanger测序证实先证者VPS33B基因杂合突变 **注** PPP：贫血小板血浆；PRP：富血小板血浆

**表1:** VPS33B基因变异家系致病突变和临床特征

家系成员	性别	年龄（岁）	与先证者关系	突变/杂合性	VWF∶Ag（%）	症状
Ⅱ-1	男	81	爷爷的表哥	c.869G>C/Het	56	无明显症状
Ⅱ-2	男	77	爷爷的大哥	–	120	–
Ⅱ-3	男	75	爷爷的二哥	c.869G>C/Het	48	偶有皮肤淤青
Ⅱ-5	女	71	奶奶	–	112	–
Ⅲ-1	男	55	Ⅱ-1之子	c.869G>C/Het	45	年轻时鼻出血
Ⅲ-2	男	59	大伯	–	110	–
Ⅲ-3	女	55	姑妈	c.869G>C/Het	39	皮肤淤青
Ⅲ-4	男	57	父亲	c.869G>C/Het	50	无明显症状
Ⅲ-5	女	57	母亲	–	90	–
Ⅲ-6	女	50	姑妈	–	83	–
Ⅳ-1	女	29	堂妹	c.869G>C/Het	52	偶见皮肤淤青
Ⅳ-2	女	31	先证者	c.869G>C、1474 dup A/Het	2	皮肤淤青，月经量增多
Ⅳ-3	男	40	丈夫	–	115	–
Ⅴ-1	男	2	儿子	–	98	–

**注** Het：杂合子；VWF∶Ag：血管性血友病因子抗原

**图2:**
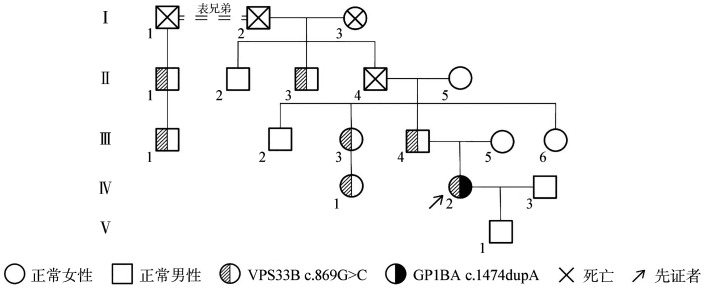
VPS33B基因变异家系图

患者围产期给予输注新鲜血浆治疗，将VWF∶Ag和FⅧ∶C提高到10％以上，孕38周经剖宫产顺利分娩一健康男婴，产后恢复良好。男婴各项凝血因子及凝血功能正常，基因测序未检出携带VPS33B基因及GP1BA基因变异。

## 讨论并文献复习

VPS33B是转运蛋白家族成员之一，主要存在于内皮细胞和血小板内。它与VPS16、VIPAS39等蛋白组成复合物，参与多种蛋白的胞内转运和分泌。VPS33B与伴侣分子组成的复合物在胞质内通过Sec-1样结构域与SNARE（可溶性N-乙基马来酰亚胺敏感因子附着蛋白受体复合物）结合，参与囊泡与细胞膜之间的融合，从而介导囊泡内的各种蛋白向细胞外分泌[6]。纯合VPS33B基因缺陷可导致ARC综合征，该症为常染色体隐性遗传，临床上极罕见，由于肝脏细胞、肾脏细胞等囊泡转运功能受损，导致患者出现关节挛缩、肾功能不全和胆汁淤积等临床表现[7]–[9]。本研究中的先证者肝肾功能正常、无骨关节畸形、身体发育正常，不存在ARC综合征的临床表现，同时应用各种测序技术也未发现除R290P杂合变异外的其他VPS33B基因变异，因此从理论上也排除了该患者为ARC综合征的可能。

研究显示，VPS33B在血小板内参与α颗粒的转运[5],[10]。由于血小板α颗粒内包涵多种黏附分子和凝血成分，因此理论上VPS33B缺陷可能影响α颗粒内各种蛋白的分泌，其中就包含VWF分子。Dai等[11]研究显示，VPS33B缺乏可影响巨核细胞内囊泡形成，进而影响VWF分子的转运。但在临床中，未见由VPS33B基因变异导致VWD的报道，这可能是由于血浆中的VWF不仅来源于血小板和内皮细胞内的α颗粒，还来源于血小板W-P小体。W-P小体由VWF分子的超大聚合物集聚而成，其本质并不是囊泡，因此其分泌机制不依赖于VPS33B。这也是临床上ARC综合征患者并无严重VWF降低的原因。另一方面，杂合VPS33B基因变异引起的血浆VWF降低在很多情况下并未达到确诊VWD的水平且临床症状轻微，因此大部分携带者并没有进行深入的基因检测。本例家系的发现，是由于先证者同时合并GP1BA基因变异导致循环中VWF水平进一步降低，才引起临床医师的关注，进而对先证者及其家系成员开展进一步的基因检测和临床特征分析，通过家系分析各成员VPS33B基因与血浆VWF水平，我们发现c.869G>C变异与携带者VWF水平显著相关。进而通过分析c.869G>C变异引起的氨基酸改变R290P对VPS33B蛋白结构的影响，显示R290P可干扰VPS33B与配体之间的结合，从而可能影响VPS33B转运复合物对囊泡的转运，使R290P变异携带者的血小板和内皮细胞分泌VWF的功能受限，从而出现不同程度的血浆VWF水平降低。

GP1BA基因编码GPⅠbα，GPⅠbα在血小板表面介导VWF与血小板之间的黏附，GPⅠbα-VWF间的结合在生理性止血过程和病理性血栓形成过程中都有重要作用[12]。GP1BA基因某些位点的变异可导致GPⅠbα与VWF分子间的结合力增强，从而使循环中的VWF被吸附到血小板表面，导致血浆VWF水平降低[13]，这类患者的血浆VWF水平大都在40％～70％，未见VWF严重降低的报道。同时，由于血小板表面黏附的VWF在血管损伤局部依然可以发挥止血作用，因此患者尽管VWF水平低于正常，但并不出现自发出血症状，这一特征与本研究中的先证者临床表现相符合。文献中把这类患者称为“假性VWD”[14]。该患者检出GP1BA基因杂合变异c.1474dupA，变异率为41％。ClinVar数据库中收录有该变异与“血小板型假性VWD”相关性的报道，美国遗传学会（ACMG）将该变异导致“血小板型假性VWD”的致病可能性定义为5级中的第2级（Likely Pathogenic）。由于通常认为“血小板型假性VWD”为常染色体隐性遗传病，并且该患者的临床症状与“血小板型假性VWD”也不完全相符（患者未出现血小板数量减少），因此尚不能将该先证者确诊为“血小板型假性VWD”。先证者母亲未检出该变异，因此我们认为先证者的这一变异为新发，同时也存在不同胚层变异嵌合的可能性。先证者的儿子没有遗传其母亲VPS33B基因和GP1BA基因中的杂合变异，其血浆VWF水平为98％，亦无其他临床症状。

本研究首次报道VPS33B基因杂合变异引起VWF水平降低的家系，也是首次报道VPS33B基因联合GP1BA基因双重杂合变异引起重度VWF水平降低，这对于理解和诊断临床上未检出VWF基因变异的部分VWD患者有重要意义。
